# Combined Predictive Value of Serum Procalcitonin and Platelet Indices in Differentiating Gram-Negative and Gram-Positive Bloodstream Infections

**DOI:** 10.7759/cureus.91390

**Published:** 2025-09-01

**Authors:** Vibha Sharma, Shubhransu Patro, Arushi Choudhary, Sujit Pradhan, Basanti Pathi

**Affiliations:** 1 General Medicine, Kalinga Institute of Medical Sciences Bhubaneswar, Kalinga Institute of Industrial Technology (KIIT) Deemed to be University, Bhubaneswar, IND; 2 Critical Care Medicine, Kalinga Institute of Medical Sciences Bhubaneswar, Kalinga Institute of Industrial Technology (KIIT) Deemed to be University, Bhubaneswar, IND; 3 Microbiology, Kalinga Institute of Medical Sciences Bhubaneswar, Kalinga Institute of Industrial Technology (KIIT) Deemed to be University, Bhubaneswar, IND

**Keywords:** gram-negative bacteria, gram-positive bacteria, platelet indices, procalcitonin, sepsis

## Abstract

Background: Sepsis is caused by bloodstream infections (BSIs) and potentially results in death. Parameters like platelet (PLT) indices and procalcitonin (PCT) are common inflammatory markers used for the diagnosis and assessment of sepsis. Thus, the present study aimed to explore the predictive value of combined serum PCT and PLT indices to differentiate the host responses of Gram-positive bacteria (GPB) and Gram-negative bacteria (GNB) in BSIs.

Materials and methods: A prospective cohort study was conducted at the Critical Care and General Medicine Department of Kalinga Institute of Medical Sciences, a tertiary care hospital in Bhubaneswar, Odisha, India, by including 385 BSI-diagnosed patients consecutively over the period of two years. All patients were more than 18 years of age and had culture-positive sepsis diagnosed based on the sepsis-3 criteria. Laboratory investigations such as complete blood count, serum PCT, and bacterial cultures for GPB and GNB, were done and correlated with clinical features. In statistical analysis, univariate and multivariate analyses (logistic regression analysis) were done, and a multivariate regression model was proposed by combining PCT and PLT indices for both Gram-positive and Gram-negative BSI cases.

Results: BSI cases were confirmed from culture-positive. Out of 385 BSI cases, 51.9% were Gram-negative and 48.05% were Gram-positive. *Escherichia coli *and *Staphylococcus aureus *were the most common organisms. A significantly higher level was observed in hematological parameters such as white blood cell count (WBC) (18.02 cells/µL ± 9.06) and PLT indices; mean platelet volume (MPV) (12.24 fL ± 2.02), PLT (159.11 x 10^3 ^mcl ± 76.7); and platelet distribution width (PDW) (14.13 fL ± 13.03) in GNB. Elevated levels were also observed in PCT (37.20 ± 40.4 µg/L), a key biomarker for bacterial infection and sepsis. Generalized linear model (GLM) based on the combined value of the above significant hematological parameters demonstrates a strong ability to discriminate GNB infection, with an area under the curve (AUC) of 0.8644. The optimal threshold for this model is 0.433, which provides a balanced sensitivity and specificity, both at 0.80. The high value of the AUC indicates the potential utility of the GLM-based prediction tool to classify the GNB infection.

Conclusion: A multi-biomarker linear model, by combining PCT, WBC, MPV, PDW, and PLT-derived ratios, can effectively differentiate Gram-negative from Gram-positive BSIs. Elevated PCT, WBC, and PLT indices indicate Gram-negative infections.

## Introduction

Worldwide, bloodstream infections (BSIs) are more prevalent as the cause of sepsis and septic shock, with a high mortality rate ranging from 15% to 30% [1]. Although there has been a decline in the incidence and mortality rates of sepsis over the past three decades, the mortality rate remains above 15% [2-4]. Sepsis is primarily characterized by a dysregulated host response to infection, resulting in organ dysfunction. The immune system typically combats pathogens by triggering an inflammatory response upon microbial invasion [1]. However, in sepsis, this response becomes systemic and relentless, leading to irreversible tissue damage and mortality. The development of sepsis and septic shock involves a complex interplay between bacterial components (such as the cell wall and secreted products) and host response actions. The exact pathogenicity of sepsis remains a subject of debate. A major challenge is to differentiate between Gram-negative and Gram-positive bacteremia. In general practice, Gram-positive bacteria (GPB) and Gram-negative bacteria (GNB) are differentiated by a staining method based on the reaction of dye with the cell wall and bacterial morphology.

Precise identification of the bacterial pathogen responsible for sepsis requires culture-based methods, which typically take time to yield results. This delay often necessitates the use of empirical antibiotic therapy based on local guidelines or antimicrobial stewardship programs. The choice of empirical antibiotics usually covers a broad spectrum of pathogens until culture results and susceptibility data become available, allowing for more targeted therapy.

Several studies have suggested that infection with GPB elicits a more robust inflammatory response compared to GNB [5]. Blood parameters like hemogram parameters and a key biomarker, procalcitonin (PCT), are useful auxiliary tools in the diagnosis and assessment of sepsis, but their ability to rapidly differentiate bacterial classifications or guide empirical antibiotic choice remains unclear. Elevated PCT levels can indicate the presence of bacterial infection, but individually, they cannot reliably distinguish between Gram-negative and Gram-positive infections, and the specificity and sensitivity have not yet been established [6]. In particular, PLT and MPV have proven useful as indicators of inflammatory activity and treatment efficacy in infectious diseases [7]. MPV, which reflects platelet (PLT) activation, has been linked to a variety of inflammatory and infectious disorders, including hepatic hydatid cysts, tuberculosis, chronic prostatitis, nasal polyposis, rheumatoid arthritis, obesity, coronary artery disease, type 2 diabetes mellitus, and irritable bowel disease [8-13]. Platelet distribution width (PDW) has also been associated with several conditions, such as irritable bowel syndrome, diabetes mellitus, coronary artery disease, and tuberculosis. Studies have shown increased mean platelet volume-to-platelet count ratios (MPV/PLT) in individuals diagnosed with type 2 diabetes mellitus, severe sepsis, and influenza A infection [14-16]. This evidence suggests that PLT-associated metrics could serve as valuable biomarkers in the context of inflammatory and infectious diseases. 

A comprehensive understanding of the prevalence, causative agents, and antibiotic resistance patterns of bacterial BSIs in India is lacking a clear information on the type of bacteria. This information is crucial for healthcare providers to enhance empirical treatment approaches and ensure timely antimicrobial therapy. In addition, it is vital to identify and monitor the sources of BSIs in order to prioritize and implement preventive measures effectively.

Here, quantitative research reveals the pathogen classification in sepsis and aims to explore the correlation of blood parameters with GPB and GNB for therapeutic action. It also aims to address the gap by testing a combined biomarker model with PCT, PLT indices, and hematological indices and to propose an effective model for early infection stratification. 

## Materials and methods

Study settings and design

A pathogen-based survey, prospective cohort study in BSIs was conducted at the Critical Care and General Medicine Department of Kalinga Institute of Medical Sciences, a tertiary care hospital in Bhubaneswar, Odisha, India, over a period of two years (2022-2024), with ethical approval (IEC NO: KIIT/ KIMS/ 965/2022/ Dt. 18-07-2022). All BSI consecutive patients (n = 385) diagnosed from culture reports during the study period were included with consent.

Study procedure

Screening, Sample Collection, and Laboratory Investigations 

**Figure 1:**
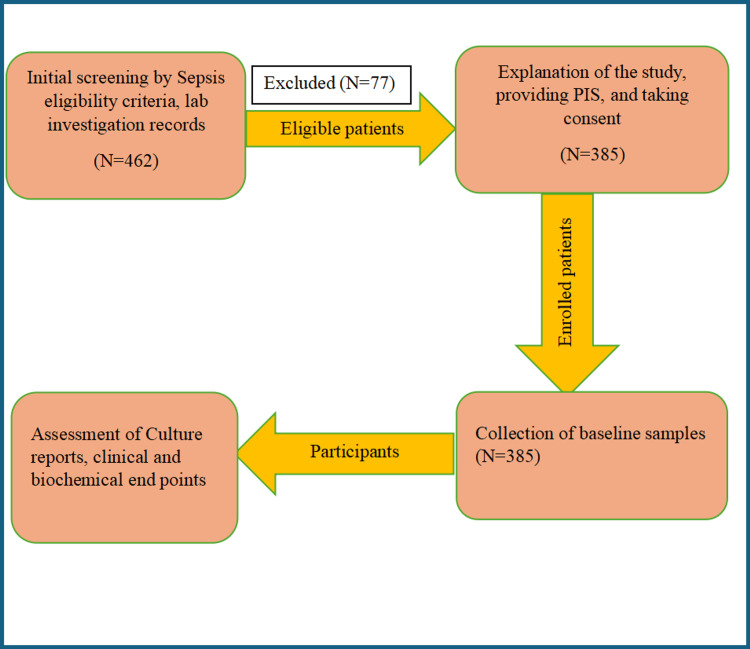
Flow chart of bloodstream infection (BSI) patient inclusion as per the inclusion and exclusion criteria

Before enrolment into the study group, all the patients were screened with a complete medical history. The patient inclusion criteria were age 18 years and above and culture-positive sepsis diagnosed based on the 2016 sepsis-3 guidelines (organ dysfunction as identified by an increase of two points in sequential organ failure assessment (SOFA) score). Patients who were previously diagnosed with blood disorders, malignant conditions, or autoimmune diseases and patients on treatment with anticoagulants, anti-PLT agents, hemostatic agents, granulocyte boosters, or clinical transfusion therapy (e.g., PLTs, red blood cells, or plasma) were excluded (Figure 1).

The blood sample was collected and cultured for the diagnosis of Gram-positive and Gram-negative infections. The bacterial streaking method was applied for the confirmation of GPB and GNB. At the same time, laboratory investigations such as complete blood count and serum PCT were estimated to correlate clinically and other common investigations like urine culture. Sputum examinations were done to localize the site of infection responsible for disease severity. Hematological parameters were estimated by an automated analyzer, and a chemiluminescent immunoassay was used for quantitative determination of the PCT level in human serum by Beckman Coulter. All demographic features and clinical symptoms were recorded in the ethically approved case record proforma for statistical analysis. 

Statistical Analysis

Statistical analysis was performed using R version 3.6.2 for continuous variables. The results are presented in mean and standard deviation, while categorical data are expressed as proportions. The Mann-Whitney U test was applied for median value comparison, and the t-test was applied for mean value comparison. All categorical data were analyzed through Fisher's exact test. Binary logistic regression analysis was applied for predicting the likelihood of GNB and GPB infection by using hematological and biochemical parameters as independent variables. Binary outcome variables are the presence of GNB were GPB (1/0), and the link function logit (log-odds) was used for the calculation. The study used a significance threshold of p < 0.05, and a receiver operating characteristic (ROC) curve was constructed to evaluate the relationship between sensitivity and specificity.

## Results

**Figure 2:**
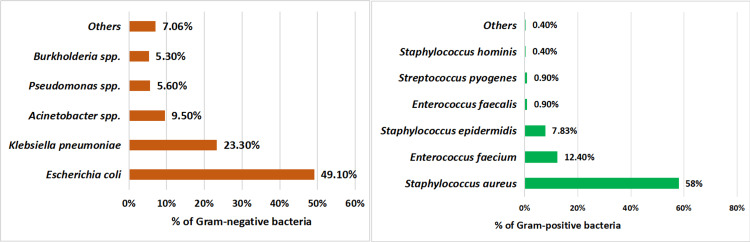
Classification of Gram-negative and Gram-positive bacteria in bloodstream infection patients

A total of 385 culture reports revealed 200 GNB and 185 GPB, which were detected from cultures of blood, urine, and sputum samples (Figure 2).

The classification of GPB and GNB shows that *Escherichia coli* was the most prevalent in GNB, accounting for 139 cases (49.1%). Among GPB, *Staphylococcus aureus *was the most commonly isolated organism, detected in 127 cases (58%).

Table 1 shows the demographic features of the study population. The mean age of BSI patients in both groups varied between 57.57 and 60.77 years. In terms of gender distribution, male patients were prevalent in comparison to females in both groups.

**Table 1:** Clinico-demographic features of Gram-positive bacteria (GPB) and Gram-negative bacteria (GNB) bloodstream infection patients

Characteristics	GNB group (n = 200)	GPB group (n = 185)
Age (mean ± SD)	60.77 ± 13.02	57.57 ± 12.23
Gender		
Male	124 (62%)	115 (62.1%)
Female	76 (38%)	70 (37.83 %)
Comorbidities		
Hypertension	77 (38.5%)	59 (32.43%)
Diabetes	87 (43.5%)	69.9 (37.83%)
Other comorbidity	46 (23%)	51.48 (28.6%)
Lifestyle		
Smoking	34 (17%)	43.84 (23.7%)
Alcoholism	28 (14%)	19.98 (10.8%)
Type of sample		
Blood	200 (100%)	185 (100%)
Urine	58 (29%)	24 (12.97%)
Sputum	20 (10%)	4(2.16%)
Other	5 (2.5%)	4(2.16%)

Regarding comorbidities, 38.5% of patients in the GNB group had hypertension, compared to the GPB group (32.43%). The prevalence of diabetes was 43.5% in the GNB group and 37.83% in the GPB group. Other comorbidities were reported by 23% of patients in the GNB group and 28.6% in the GPB group. Among all major lifestyle factors are smoking and alcoholism. 

Comparative hematological and biochemical parameters among GPB and GNB are explained in Table 2. The level of CRP was significantly higher in GNB- than in GPB-infected sepsis patients. All the hematological parameters, such as PCT, PLT indices, WBC, neutrophil, monocytes, and MPV/PLT, are significantly higher in GNB- in comparison to GPB-infected cases.

**Table 2:** Comparative analysis of hematological parameters and calculated indices in both groups

Parameters	GNB (n = 200) (mean ± SD)	GPB (n = 185) (mean ± SD)	P-value (t-test)
C-reactive protein (CRP) (mg/dL)	152.95 ± 82.2	113.15 ± 110.55	<0.001
Procalcitonin (µg/L)	37.20 ± 40.4	9.93 ± 31.7	<0.001
Mean platelet volume (MPV) (fL)	12.24 ± 2.02	10.28 ± 1.66	0.019
Platelet distribution width (PDW) (fL)	14.13 ± 13.03	12.07 ± 2.1	<0.001
Platelet (PLT) (10³ μL )	159.11 ± 76.7	217.25 ± 90.19	0.016
White blood cell (WBC) (cells/μL)	18.02 ± 9.06	14.26 ± 4.2	0.032
Lymphocyte (µL)	8.89 ± 8.8	11.67 ± 7.5	0.016
Neutrophil (cells/μL)	85.98 ± 8.5	81.09 ± 9.0	0.012
Monocyte (%)	2.95 ± 1.76	3.76 ± 2.1	0.019
MPV/PLT	0.12 ± 0.25	0.05 ± 0.02	0.014
Systemic Immune inflammation index (SII)	3183.89	2606.79	
PDW/PLT	0.20	0.06	0.012
PLT/PCT	46.31	413.73	<0.001
PLT/PCT	264.40	2762.46	
PDW/PCT	4.7	17.9	<0.001
MPV/PCT	3.98	16.1	<0.001

Correlation of hematological parameters with calculative indices revealed that CRP is not significantly associated with the outcome group. Its coefficient indicates no strong evidence that CRP levels differentiate between the GPB and GNB groups. The levels of PCT, MPV, and PDW are significantly increased by 0.321, 0.2467, and 0.103, respectively, in GNB than GPB (Table 3).

**Table 3:** Correlation analysis of haematological parameters and calculated indices

Parameters	Estimate	P-values	Z-values	Std. error	95% confidence interval
Procalcitonin	0.32116	3.97e-06	4.6130169	0.006	(0.30936, 0.33296)
Mean platelet volume (MPV)	0.246701	0.0225	2.2819134	0.108	(0.03493, 0.45847)
Platelets	-0.081386	0.036	0.001433136	0.967	(-1.98663, 1.82386)
Platelet distribution width (PDW)	0.103060	0.041526	0.072044297	1.430	(-2.70064, 2.90676)
White blood cell (WBC)	0.087002	0.01163	3.2481506	0.026	(0.03611, 0.13789)
Lymphocyte	0.098724	0.0648	0.053468170	1.846	(-3.51926, 3.71671)
Neutrophil	0.118861	0.0163	2.4020941	0.049	(0.02286, 0.21486)
Monocyte	0.025753	0.7507	0.81069	0.317	(-0.59556, 0.64707)
Systemic immune-inflammation index (SII)	7.42	0.0359	6.9217	3.536	(0.489, 14.351)
MPV/PLT	7.30	0.0405	3.565	2.048	(3.285, 11.315)
PDW/PLT	0.3460	0.046171	0.4999285	6.921	(-13.220, 13.912)
MPV/PCT	-0.1874	0.01093	1.6011277	1.170	(-2.481, 2.106)
PDW/PCT	-0.1597	0.0316	2.1491918	7.432	(-14.717, 14.397)
PLT/PCT	-0.039	0.051	1.922	2.02	(-4.007, 3.929)

Mean PLT values are negatively correlated with clinical outcomes in GNB than GPB. WBC is significantly associated with the clinical outcomes; for each unit increase in WBC, the log odds of being in the GNB group increase by 0.0870. The lymphocyte count is marginally significant. For each unit increase in the lymphocyte count, the log odds of being in the GNB group increase by 0.0987. Neutrophil count is significantly associated with the outcome group. For each unit increase in neutrophil count, the log odds of being in the GNB group increase by 0.1189. This suggests that higher neutrophil counts are associated with higher odds of being in the GNB. Hematological parameters such as PCT, MPV, WBC, PDW, and neutrophil are significantly associated with the disease outcome, indicating that higher values are associated with the GNB group.

Among calculative indices, the systemic immune-inflammation index (SII) exhibited a significant difference between the GNB and GPB groups, potentially serving as a distinguishing factor in GNB. The mean MPV/PLT ratio is significantly reduced in GPB-infected cases than GNB cases, considered as a potentially important marker to distinguish between the GNB and GPB groups. Similarly, the PDW/PLT ratio is higher in GNB groups than in GPB groups. This significant disparity further suggests the PDW/PLT ratio as a relevant parameter for distinguishing between these groups. The ratio of PLT/PCT revealed maximum levels (413.0) in the GPB group in comparison to GNB-infected cases. This high value of the ratio rules out GNB. MPV/PCT ratio is also higher in GPB infections. Lastly, the PDW/PCT ratio was higher (17.9) in GPB infections in comparison to GNB (4.7).

Multivariate regression analysis predicts a model equation for GNB infection severity by analyzing MPV, SII, MPV/PLT, PWD/PLT, PCT, and PDW.

GLM based on the combined value of significant hematological parameters demonstrates a strong ability to discriminate GNB infection, with a high value of AUC, i.e., 0.8644. The high value of AUC indicates the potential utility of the GLM-based prediction tool to classify the GNB infection. 

The equation for GNB infection severity is:

logit(GNB) = 1.114 + 0.24 * MPV + 7.42 * SII + 7.30 * (MPV/PLT) + 0.34 * (PDW/PLT) + 0.32 * Procalcitonin + 0.103 * PDW

The equation for GPB infection severity is:

logit(GPB)=6.335+(0.039×PLT/PCT)+(0.1874×MPV/PCT)+(0.1597×PDW/PCT)+(0.081386×Platelets)

The combined model equation presented for both GNB and GPB follows a binary logistic regression. The models predict the likelihood of GNB and GPB infection severity based on a set of continuous predictors. The equation is based on the components of the Y axis (binary - presence of GNB (coded as 1) vs. GPB (coded as 0) and components of the X axis (independent significant variables like MPV, SII, ratios of MPV/PLT, PDW/PLT, PCT, and PDW. 

Efficacy of the predictor for severity prediction with a differentiation between GPB and GNB infection is elaborated in Table 4 and Table 5. The ROC analysis of multiple variables, along with the combined model (as analyzed by binary regression analysis), demonstrates a strong ability to discriminate between cases with and without infection, with an AUC of 0.8644. This indicates that the correctly identifies 80% of true-positive and true-negative cases in GNB infection. In the case of GPB, the AUC of 0.79 with a threshold of 0.47 has been observed with a sensitivity of 0.76 and a specificity of 0.78, indicating its robust diagnostic ability. Table 5 presents the individual AUC, sensitivity, and specificity for positive predictors of GNB and GPB infection, showcasing the performance of different hematological parameters in the diagnostic model.

**Table 4:** Individual AUC with sensitivity and specificity for positive predictors of Gram-negative bloodstream infection (GNBSI) AUC: area under the curve, CI: confidence interval, SII: systemic immune-inflammation index, MPV/PLT: ratio of the mean platelet volume and platelet, PDW/PLT: ratio of the platelet distribution width and platelet, PLT: platelet

Parameter	AUC	95% CI	Threshold	Sensitivity	Specificity
Combined model	0.86	17102.64-23067.42	0.433	0.80	0.80
MPV/PLT	0.674243	0.07-0.11	0.055	0.675	0.64
PDW/PLT	0.67196	0.06-0.21	0.065	0.69	0.637838
Procalcitonin	0.797743	20.2-28.0	12.8815	0.625	0.854054
MPV	0.640595	1.09-11.62	10.65	0.425	0.816216
PDW	0.782027	12.18-14.1	12.25	0.78	0.686487
SII	0.645068	2303.1-31.06.09	171.5	0.61	0.708108

**Table 5:** Individual AUC with sensitivity and specificity for positive predictors of GPBSI AUC: area under the curve, CI: confidence interval, MPV/PLT: ratio of the mean platelet volume and platelet, PDW/PLT: ratio of the platelet distribution width and platelet, PLT: platelet

Parameter	AUC	95% CI	Threshold	Sensitivity	Specificity
Combined model	0.79	17102.64-23067.42	0.47	0.76	0.78
PLT	0.69	171.7-195.45	171.5	0.61	0.70
PDW/PCT	0.76	2.78-13.42	0.65	0.54	0.91
PLT/PCT	0.81	29.43-44.37	14.55	0.66	0.93
MPV/PCT	0.74	23.0-17.08	0.55	0.56	0.91

AUC for individual significant predictors

**Figure 3:**
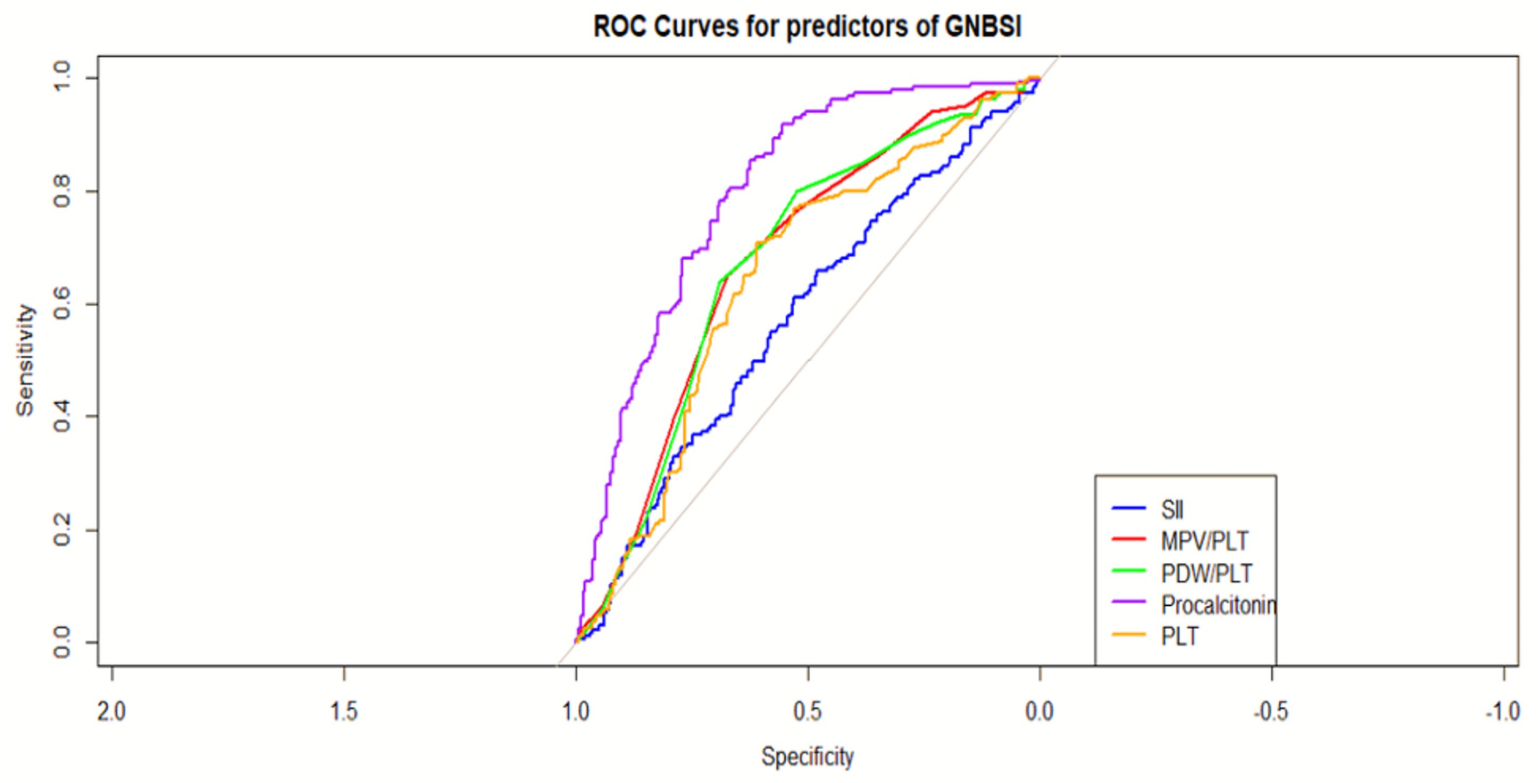
Receiver operating characteristics (ROC) of predictors for Gram-negative bloodstream infection (GNBSI) SII: systemic immune-inflammation index, MPV/PLT: ratio of the mean platelet volume and platelet, PDW/PLT: ratio of the platelet distribution width and platelet, PLT: platelet

**Figure 4:**
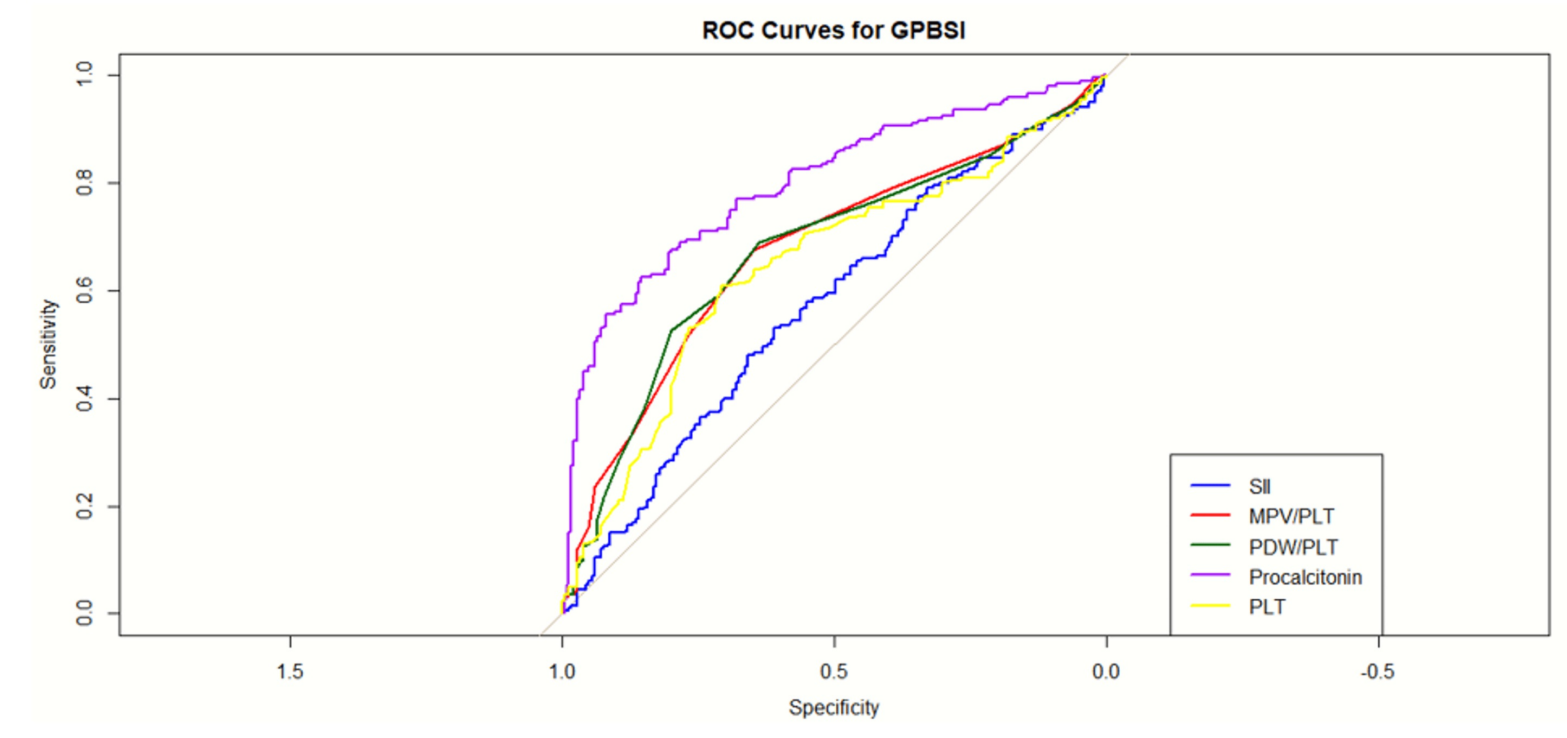
Receiver operating characteristics (ROC) of predictors for Gram-positive bloodstream infection (GPBSI) SII: systemic immune-inflammation index, MPV/PLT: ratio of the mean platelet volume and platelet, PDW/PLT: ratio of the platelet distribution width and platelet, PLT: platelet

PCT shows good discriminative ability with an AUC of 0.7977. At the threshold of 12.8 with a 62.5% true-positive rate and a high specificity of 0.8541, which indicates that a high value of PCT can help to differentiate GNB and GPB infection (Table 4). MPV has a lower discriminative power with an AUC of 0.6406. At the threshold of 10.65, low sensitivity of 0.425 (42.5% true-positive rate) but a high specificity of 0.8162 (81.6% true-negative rate). PDW has a good discriminative ability with an AUC of 0.7820, at the threshold of 12.25, a high sensitivity of 0.78 (78% true-positive rate), and a moderate specificity of 0.6865 (68.7% true-negative rate). For the MPV/PLT ratio, the AUC is 0.6742, showing moderate discriminative power. The PDW/PLT ratio has a similar performance to MPV/PLT with an AUC of 0.6720. SII has moderate discriminative power with an AUC of 0.6451 (Figure 3 and Figure 4).

## Discussion

In this study, we evaluated several haematological and biomarker parameters to distinguish between GNB and GPB infections. Our results reveal distinct differences between these two types of bacterial infections, with PCT, MPV, PDW, WBC, and neutrophil count emerging as significant predictors of GNB infection. Conversely, PLT count and various PLT-derived ratios such as MPV/PLT, PDW/PLT, and PLT/PCT demonstrated significant differences favouring GPB infections. As individual predictors, elevated levels of PCT significantly differentiate GNB from GPB BSIs. This finding aligns with previous research indicating that PCT is a reliable biomarker for bacterial infections, particularly in distinguishing between GNB and GPB.

Li et al. (2023) investigated the effectiveness of serum PCT levels and other inflammatory markers in differentiating GNB sepsis from Gram-positive bacterial and fungal sepsis [17]. Their study included 328 bacteraemia cases and found that PCT levels were significantly higher in Gram-negative sepsis (mean 7.47 ng/mL) compared to Gram-positive (mean 0.48 ng/mL) and fungal sepsis (mean 0.60 ng/mL) (P < 0.001). ROC curve analysis determined that a PCT cut-off value of 2.44 ng/mL effectively distinguished Gram-negative sepsis from Gram-positive sepsis, with 68.4% sensitivity and 77.1% specificity. The study concluded that PCT is a valuable biomarker for differentiating Gram-negative sepsis from other types of sepsis [18]. A systematic review Hoeboer SH et al. (2016) demonstrated that elevated PCT levels are associated with severe bacterial infections, with higher levels observed in Gram-negative infections as compared to Gram-positive ones [19].

Our analysis revealed that MPV was significantly higher in the GNB group compared to the GPB group (mean: 12.24 ± 2.02 vs. 10.28 ± 1.66, p-value: 0.019; 10.4 vs. 9.76, p < 0.001). These results are consistent with previous studies suggesting that increased MPV is associated with bacterial infections, including those caused by Gram-negative pathogens, as Gram-negative pathogens are responsible for a more inflammatory reaction. A study by Abe R et al stated that Gram-negative organisms induce a greater immune response and hence all the inflammatory markers are hypothesized to increase more in GNB sepsis [20].

Similarly, PDW was significantly higher in the GNB group compared to the GPB group (mean: 14.1 vs. 10.1, p < 0.001). This result is in agreement with research indicating that PDW can reflect PLT activation and inflammatory responses. A study by Dwaya A et al found that MPV and PDW, both PLT indices, are increased in bacterial sepsis, but the rise is more in GNB sepsis as compared to GPBSI [21].

In the present study, WBC and neutrophil counts were significantly higher in the GNB group than in the GPB group. These findings are consistent with other studies, which show increased WBC and neutrophil counts in response to severe bacterial infections. For example, Suwarto et al. (2017) conducted a retrospective study in Jakarta, Indonesia, to compare laboratory markers of infection in patients with Salmonella bacteraemia versus other Gram-negative bacteraemia in 61 patients. Their bivariate analysis revealed that Gram-negative bacteraemia was significantly associated with elevated leucocyte count (cut-off: 13,850/µL), neutrophil count (cut-off: 85.0%), and PCT levels (cut-off: 4.0 ng/mL), all of which were higher compared to the Salmonella group (p < 0.001 for each variable). The study concluded that these laboratory markers are significantly elevated in Gram-negative bacteraemia compared to Salmonella bacteraemia, highlighting their potential use in differentiating between these infections [22].

Among the PLT ratio indices, the GPB group had a significantly higher PLT count and ratio of PLT/PCT and PDW/PCT. This is in line with findings from studies by Beller et al. (1973) and Lin et al (2018), which demonstrated that lower PLT counts and specific PLT-derived ratios are often observed in patients with Gram-negative infections [23]. The retrospective cohort-controlled trial by Lin et al analysed the PLT levels and infectious bacteria strains in 2,308 blood culture-positive and 3,786 blood culture-negative patients from 2014 to 2016. Results indicated that thrombocytopenia (PLT < 100) with GNB is higher as compared to GPB. This study concluded that BSIs, especially those caused by GNB, significantly contribute to thrombocytopenia. Similarly, the study by Beller and Douglas (1973) found that in seven febrile patients with positive bioassays for endotoxemia, PLT counts were below 150,000/cu mm, and in four of these seven patients, blood cultures revealed Gram-negative bacteraemia. These findings suggest that endotoxemia, even in small concentrations, can directly affect PLT counts, indicating that thrombocytopenia with a normal coagulation profile in septic patients may suggest endotoxemia and Gram-negative infections [23,24]. 

In addition, our results indicate that the MPV/PLT ratio and PDW/PLT ratio are significant for distinguishing between GNB and GPB infections, which aligns with the findings of Catal F et al. (2008) where MPV and other PLT indices were shown to be differentiating indices between specific infection types, showing the utility of these ratios in differentiating between bacterial pathogens [25].

The study utilized a multivariate stepwise logistic regression model to identify the predictors for GN-BSI versus GP-BSI. The combined GLM model demonstrated robust diagnostic performance for distinguishing between GNB and GPB infections, with an AUC of 0.86 and 0.79, showing good discriminative ability [26]. The high value of AUC indicates the potential utility of the GLM-based prediction tool to classify the GNB infection. Our results reflect those of previous studies that have successfully utilized a combination of biomarkers for infection diagnosis. For instance, the study by Tsurumi A et al. (2020) highlighted the effectiveness of multi-biomarker models for diagnosing bacterial infections, further supporting the use of a composite model in a clinical setting [27]. 

In their study, Gao et al. identified that GN-BSI patients exhibited higher levels of PCT, MPV, and PDW compared to those with GP-BSI [28]. Conversely, the PLT count and related ratios like PLT/PCT were found to be lower in the GN-BSI group. The study's multivariate stepwise logistic regression analysis highlighted the independent predictive value of MPV, PWR, and PCT for GN-BSI, underscoring the potential of these markers to aid in early and accurate diagnosis.

In comparison, individual parameters like PCT and PDW showed good discriminative power, as supported by similar studies demonstrating the effectiveness of these markers in early infection diagnosis by GNB. The AUC values for individual predictors such as PCT and PDW align with findings from a study by Pociute A et al., which confirmed the role of these biomarkers in infection diagnostics [29].

The limitations of the study are single-center, lack of external validation, missing comorbidity adjustment, and possible confounding factors such as ICU versus ward patients and prior antibiotics. Multicentric validation and inclusion in sepsis scoring tools are the future direction for better conclusive findings.

## Conclusions

Elevated PCT levels, along with increased MPV, PDW, WBC, and neutrophil count, are significantly associated with Gram-negative infections. Conversely, higher PLT count and specific PLT-derived ratios such as MPV/PLT, PDW/PLT, and PLT/PCT are indicative of differentiating Gram-positive infections from Gram-negative infections. In summary, the integration of PCT levels, hemogram parameters, and specific PLT indices into a multi-biomarker diagnostic model provides a powerful tool for the early and accurate differentiation of bacterial BSIs. The multivariate GLM employed in our study exhibited strong diagnostic performance, with AUC values of 0.86 and 0.79 for Gram-negative versus Gram-positive infections. 
